# Pharmacists' knowledge of drug food administration and their appropriate patient counseling a cross-sectional study from Palestine

**DOI:** 10.1186/s41043-023-00444-9

**Published:** 2023-09-14

**Authors:** Murad Abualhasan, Shahd Tahan, Roa’a Nassar, Maysoon Damere, Hadeel Salameh, Hiba Zyoud

**Affiliations:** Department of Pharmacy, Faculty of Medicine and Health Sciences, An-Najah National University, P.O. Box 7, Nablus, State of Palestine Palestine

**Keywords:** Pharmacists knowledge, Drug-food interactions, Community pharmacists, Patient counseling

## Abstract

Pharmacists have a responsibility in the treatment of patients. Interactions between food and drugs may lead to a loss of therapeutic effectiveness or drug toxicity. Our study's objectives were to assess pharmacists' knowledge of patient counseling with regard to informing the patient about taking the drug in relation to food, as well as community pharmacists' knowledge of any pharmaceutical instructions that must be given to patients when delivering the drug. The pharmacists were assessed using an interview questionnaire. The results showed a variation in the pharmacist's knowledge about the administration of the drug with food. The chief pharmacists had better knowledge of the proper food administration counseling than that of assistant pharmacists; the percentage of those who did not give the proper food counseling was 24 vs. 58%, respectively (*P* < 0.05). Only (21%) of pharmacists with more work experience provided proper counseling, while only 18.2 and 18.7% of pharmacists with moderate and low work experience provided proper counseling, respectively. The study showed the pharmacists’ limited knowledge of drug administration with food and outdated counseling information. It is highly recommended that a continuous education system be encouraged and enforced by the ministry of health to update pharmacist’s knowledge of dispensed drugs.

**Supplementary Information:**

The online version contains supplementary material available at 10.1186/s41043-023-00444-9.

## Introduction

Pharmacists have a significant role in treatment in society, as pharmaceutical treatment requires their direct communication with patients [1–3]. Patient counseling enables pharmacists to enhance the therapeutic outcomes of patients [4]. There is evidence that effective counseling with patients requires pharmacists to update their knowledge through continuous education [5, 6]. Additionally, updating counseling knowledge is important for understanding drug-related concerns such as non-adherence, adverse reactions, and misuse [7–10]. Pharmacists are responsible for drug therapy as they can avoid and manage drug-related problems by obtaining intensive and continuous education and training [11–13]. They must have a unique knowledge base to make better decisions about the treatment of patients and educate them before and during treatment [14–16]. Further, they also play an important role as healthcare practitioners in improving access to health care and closing the gap between the future benefit of medications and the value realized [17, 18]. Because of the increasingly dynamic and varied nature of pharmacists’ position in the health care system and public health, their integrity as health care professionals with the latest skills and expertise is expected to be continuously maintained [19, 20]. Many factors—including the effects of food on gastrointestinal physiology and physicochemical interactions among medications, drug dosage forms, and dietary components—are the net product of the observed effects of food on drug absorption [21]. Various medications must be taken before, with, or after food to avoid side effects such as nausea, vomiting, and stomach irritation. Interactions between food and drugs may decrease or increase the drug effect and hence change drug bioavailability [22]. Some foods cause chelation, which affects absorption, and some foods have direct interactions with drugs. Acetylcholine, clodronic acid, didanosine, etidronic acid, penicillamine, and tetracycline often chelate with food or dairy products, or the drug directly interacts with certain food components [23]. Additionally, the physiological response to food intake, particularly gastric acid secretion, may reduce the bioavailability of certain drugs. Gastric hypersecretion and insufficient intestinal mucosa contact time in short small bowel patients reduce drug absorption, including omeprazole [24, 25].

The severity of interactions between food and drugs may be affected by the form of food and interval between eating and drug administration. Interactions between food and drugs may lead to a loss of therapeutic effectiveness or drug toxicity. The route of drug administration and the pattern of drug delivery may have a major effect on the magnitude and time course of drug action [26].

There are a variety of reasons why medicine should be taken with a meal or nutrient beverage [27]. Clinical personnel in hospitals and assisted living facilities may find it more convenient to administer a drug when inpatients receive meals [28]. Outpatient compliance with the recommended drug dose schedule may be enhanced by prescribing medication at mealtimes. Some medications irritate the stomach and intestines, and when administered with food or a nutrient beverage, the effect is lessened compared to when they are administered alone with water [29].

Studies done in Jordan and Saudi Arabia demonstrated that pharmacists had unsatisfactory knowledge about common food drug interactions, with no significant difference between hospital and community pharmacists [30, 31]. Moreover, an earlier study about the patient's perception of the Palestinian pharmacist and the need for patient counseling were performed in Palestine demonstrating high trust toward pharmacist [32, 33]. To address pharmacists' limited understanding of food-drug interactions, it is essential to enhance their education, provide continuous training through various mediums, develop accessible resources for quick reference, utilize case studies for practical application, establish clear guidelines, implement quality assessments, ensure ongoing knowledge updates, and encourage collaboration with industry experts, all of which collectively aim to improve pharmacist knowledge and ultimately enhance patient safety and care.

The objective of this study was to evaluate the pharmacist's knowledge of food administration instructions and the need to counsel the patient when taking the drug. To our knowledge, this is the first study of its kind to be conducted in Palestine, in which the pharmacist counsels patients about administering drugs with food and the important instructions that must be given to patients when the drug is dispensed. The study is significant because it reflects the situation in Palestine and the neighboring meditation countries in general.

## Methods

### Study design and setting

A cross-sectional study was conducted in the northern and middle parts of the West Bank, Palestine. The survey used a convenient sample size of 100 pharmacists working in community pharmacies in the northern and middle parts of Palestine, which has five governorates: Nablus, Qalqiliah, Jenin, Tulkarm, and Ramallah. Data were collected for over six weeks, from December 9, 2021, to January 20, 2022. The selected pharmacies were included based on all pharmacist qualifications (undergraduate and postgraduate). The selected pharmacies were from the main cities and rural areas of Palestine. Chief or assistant pharmacists who were present during the interview were handed the questionnaire; on average, the interview questions lasted around 30 min.

### Sample size

The sample size for this study was determined through a convenient method. Taking into consideration the latest available statistics, which indicated a total of 800 community pharmacy shops in Palestine, we chose to focus on five major cities that represent the northern, central, and southern regions of the West Bank. Subsequently, we randomly selected 20 pharmacies from each of these selected cities for inclusion in our study [34].

### Inclusion and exclusion criteria

In the study, we included pharmacists regardless of their age and year of graduation. All participants provided informed consent. Moreover, individuals with a diploma degree who were working in community pharmacies at the time of the interview were excluded from the study.

### Data collection instrument

In order to assess the pharmacists' knowledge, a multiple-choice questionnaire was constructed by retrieving and highlighting key topics from relevant publications. Pharmacists skilled in community pharmacy practice and patient counseling conducted the interviews.

The content validity of the questionnaire was reviewed and validated by experts who understand the investigated topic, they have read the questionnaire and made the necessary corrections to make sure that the questions effectively capture the topic under investigation. The questionnaire was tested for its internal consistency using Cronbach’s alpha (CA) for it validity and reliability.

During each visit, we interviewed only one pharmacist per community pharmacy, regardless of the presence of multiple pharmacists during the interview. Further, the questionnaire contained sociodemographic characteristics (e.g., age, gender, and level of education).

The questionnaire was made with clear goals and objectives in mind. It collected the right information and made sure that each question was clear, specific, and met the goal. It was designed to evaluate pharmacists’ knowledge on how to administer medicines, with or without food, and to examine whether they provided appropriate counseling to patients. The questionnaire included 20 questions. Approximately 20 pharmacies were included from each region; 100 pharmacists were interviewed. We classified pharmacists as having sufficient knowledge if their score was above 70%. This categorization is based on the Palestinian Pharmacy Association license examination, where the pharmacist will be given the practice license if they pass the exam with a grade of at least 70%. Finally, we compared the pharmacists’ answers and their patient counseling, with different variables, including experience, level of education, and gender.

### Statistical analysis

Data were analyzed using Statistical Package for the Social Sciences (SPSS) version 21. We conducted a descriptive analysis of the characteristics and questions. Frequencies and percentages were calculated for categorical variables and means and standard deviations and/or medians were calculated for numerical variables. Pearson Chi Square test was performed to test statistical significance.

### Ethics approval

The protocol of this study was approved by the Institutional Review Board (IRB) committee at An-Najah National University. A verbal consent was obtained from the community pharmacists who participated in the study. The informed verbal form of consent was approved by (IRB) committee at An-Najah National University.

## Results and discussion

### Descriptive analysis

The validity of the questionnaire was evaluated using the Cronbach test, which produced a Cronbach's alpha (CA) value of 0.85, which indicates that the questionnaire has a high degree of reliability.

Table 1 is a summary of the descriptive results. Around two-thirds (75%) of the pharmacists interviewed were females. This reflects the reality of community pharmacy in Palestine, where the majority of pharmacists are female [32]. One-third of the pharmacists interviewed had low experience (29%), and more than one-third had high experience (38%). Pharmacists were considered to have high work experience if they worked in community pharmacies for more than five years, and those with less than one year of experience were considered to be practitioner pharmacists of low experience; otherwise, they were considered to have moderate work experience. Those with low experience were usually assistant pharmacists under the supervision of a chief pharmacist, who by law has to serve at least two years in the community of hospital pharmacy.

**Table 1:** Descriptive analysis of the interviewed pharmacists

		% Percentage
Gender	Male	25.0
	Female	75.0
Experience	Low working experience	29.0
	Moderate working experience	33.0
	High working experience	38.0
Chief	Assistant	67.0
	Chief	33.0
Education level	Bachelor (Bsc)	91
	Doctor of Philosophy (PhD)	3
	Master (Msc)	6

On average, three pharmacists worked in each community pharmacy; however, some pharmacies had 10 pharmacists. In every pharmacy visited, only one pharmacist was interviewed to avoid bias. In the study, most interviewed pharmacists were assistant pharmacists (67%). This result reflects the real situation of community pharmacies in Palestine, where most of the working pharmacists (in community pharmacies) are assistant pharmacists working under the supervision of a chief pharmacist, who is usually the owner of the pharmacy shop. Results shows that 91% of the pharmacists that were interviewed have at least a bachelor's degree in pharmacy, while the rest have a higher educational level.

### Pharmacists’ drug instruction and counseling knowledge

Each pharmacist was asked to answer 20 questions (the questions are detailed in the Additional file 1); the questions were designed to mainly test the pharmacist's latest knowledge about drug intake before or after food and also the important counseling that needs to be given for the patient, including instructions such as how to take the drug and other warnings or expected side effects that the patient must be aware of. The overall knowledge of the pharmacists regarding food administration with drugs and appropriate counseling was rated as pass or fail: A pharmacist passed if they correctly answered at least 70% of the questionnaire [35, 36]. These criteria are in accordance with the university and pharmacist associations requirements for license qualifications. The results in Table 2 illustrated the percentage of pharmacists who gave both information regarding the food intake and the necessary drug counseling for each drug and the results are shown in column under the heading (both right), the second column represent the percentage of pharmacist who gave one of the answers correctly, i.e., either the food instructions or the necessary counseling while the third column shows the percentage of patients whose both of answers were wrong. Unfortunately, the results revealed that many pharmacists do not obtain drug instructions from scientific studies or company information leaflets regarding the interval (or lack thereof) between food and drug intake, as the study result showed only 24% of the evaluated pharmacists had given the right patient the necessary counseling and the instruction of food intake with the administered drug. Pharmacists usually obtain relevant drug information for patient counseling from their senior colleagues—which could be incorrect and outdated—and generalize it for all drug classes; for example, they ask their patients to take all antibiotics before food intake and all non-steroidal anti-inflammatory drugs (NSAIDs) after food intake. In this way, crucial and unique information about certain drugs is not conveyed to patients during counseling.

**Table 2:** Summary results of the answered questions

Questions about food intake and necessary counseling of administered drugs	% Both right	% Both wrong	% One right
Q1. Metformin	19	18	63
Q2. Glibenclamide	10	32	58
Q3. Acarbose	11	40	49
Q4. Cefuroxime	14	25	61
Q5. Azithromycin	42	27	31
Q6. Ciprofloxacin	19	41	40
Q7. Fosfomycin Tromethamin	43	26	31
Q8. Naproxen sodium	18	4	
Q9. Diclofenac potassium	3	65	32
Q10. Celecoxib	10	72	18
Q11. Sulindac	10	12	78
Q12. Phenazopyridine	61	6	33
Q13. Clindamycin HCl	9	51	40
Q14. Ulipristal acetate	28	25	47
Q15. Isotretinoin	69	5	26
Q16. Spironolactone	17	38	45
Q17. Alendronate	63	14	23
Q18. Levothyroxine	29	23	48
Q19. Fluconazole	3	51	46
Q20. Fluoxetine	12	30	58

Table 2 shows the answers to question nine, which asked how up-to-date the pharmacists were on the instructions needed for diclofenac potassium. Only 3% of the pharmacists who were interviewed gave the right answer and advice, while 65% did neither.

Only 10% of the studied sample answered question 10 correctly, which was about the appropriate food intake and counseling for celecoxib. Also, the majority of the pharmacists (72%) gave wrong counseling advice for both, while 18% gave only one correct answer.

NSAIDs are one of the most commonly prescribed medicines [37]. However, many studies showed improper prescription, dispensing, and medical counseling among the health professions [38–40]. The findings of this study also show the existence of this problem among the Palestinian pharmacists, which requires updating necessary instructions when the NSAID is prescribed.

When asked about the antifungal drug fluconazole, only 3% of the pharmacists answered correctly and gave the right advice. The rest either answered both questions incorrectly or gave only one answer that was right. However, the pharmacists’ performance was better when asked about drugs such as phenazopyridine (Q12); 61% answered correctly and provided appropriate counseling, whereas only 6% answered both questions incorrectly. This result shows that more than half of the pharmacists polled correctly answered one of the drug-related questions. When we looked thoroughly at the answers to the questions about this specific drug, we found that the pharmacist correctly stated that the patient needed to be warned about the change in urine color. This information is widely shared among pharmacists in the area, and it came from a more experienced pharmacist. Unfortunately, many pharmacists world-wide have their information for senior pharmacists without getting back to the drug information from its original source [41].

**Fig. 1:**
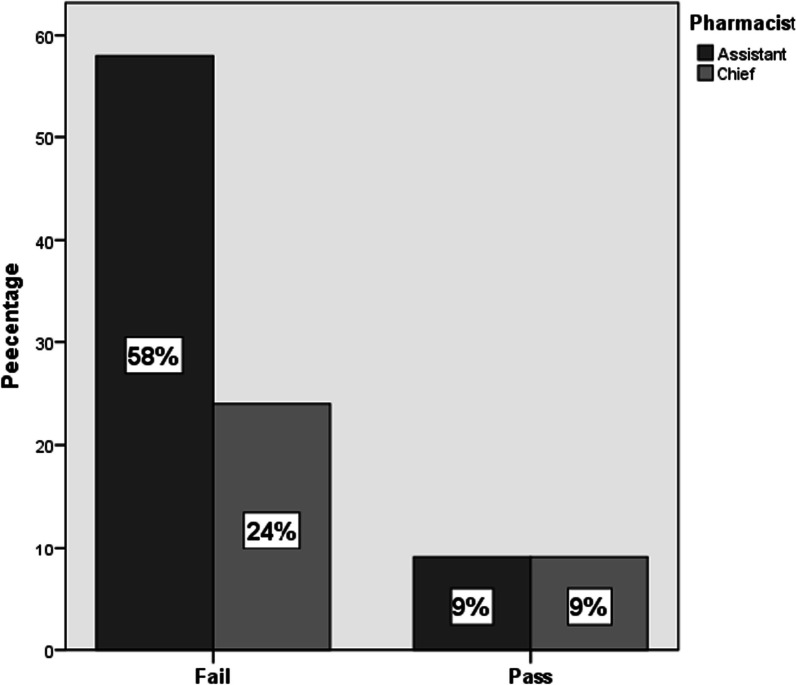
Chief pharmacist compared to assistant pharmacist providing appropriate counseling

The results in Fig. 1 showed a variation between the chief and assistant pharmacists—the chief pharmacists performed better than the assistant pharmacists in drug-food counseling where 58% of the assistant pharmacists failed to provide correct food administration counseling for the prescribed medicine compared to chief pharmacist (24%). However, Pearson Chi-square (Table 3) test showed that the significant difference was not statistically significant (*P* < 0.05). According to the low of pharmacy association a licensed pharmacist can become chief pharmacist only after a working experience of at least two years. This result clearly demonstrates that chief pharmacists who have more experience and knowledge were better in counseling. In a comparable study conducted in Lebanon, proposed measures involve providing additional training sessions and workshops to help pharmacists update their knowledge on various drug interactions. Additionally, the study suggests the adoption of software programs within pharmacies to promptly identify such interactions [42].

**Table 3:** Pearson Chi Square test result of statistical difference between chief and assistant pharmacists

Chi-Square tests					
	Value	Df	Asymp. Sig. (2-sided)	Exact Sig. (2-sided)	Exact Sig. (1-sided)
Pearson Chi-Square	1.452^a^	1	.228		
Continuity correction^b^	.724	1	.395		
Likelihood ratio	1.375	1	.241		
Fisher's exact test				.291	.195
Linear-by-linear association	1.438	1	.230		
N of valid cases	100				

^a^1 cells (25.0%) have expected count less than 5. The minimum expected count is 3.30

^b^Computed only for a 2 × 2 table

**Fig. 2:**
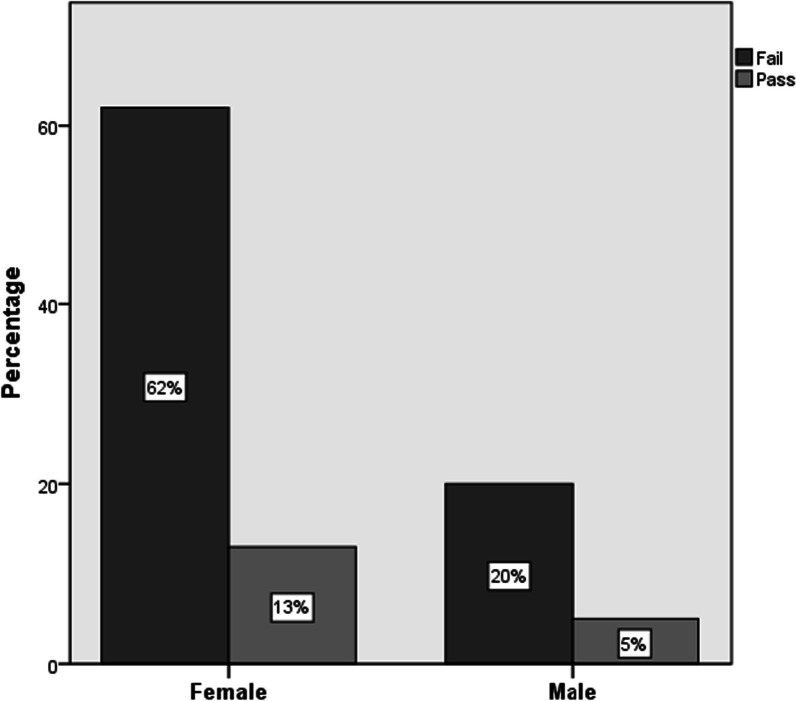
Providing appropriate counseling depending on gender

The results in Fig. 2 show that the female pharmacists scored slightly better than the male pharmacists: 18 and 17.3% of the female and male pharmacists, respectively, have a pass score. However, this difference was not statistically significant (*P* > 0.05). These results illustrate that both pharmacist genders lack sufficient and correct knowledge of drug counseling for food intake and necessary instruction for the patients. In a study involving pharmacy students, it was evident that they displayed inadequate understanding of Food-Drug Interactions and Alcohol-Drug Interactions. It suggested a need to place greater emphasis and dedication toward increasing awareness about potential FDIs and ADIs [43].

**Fig. 3:**
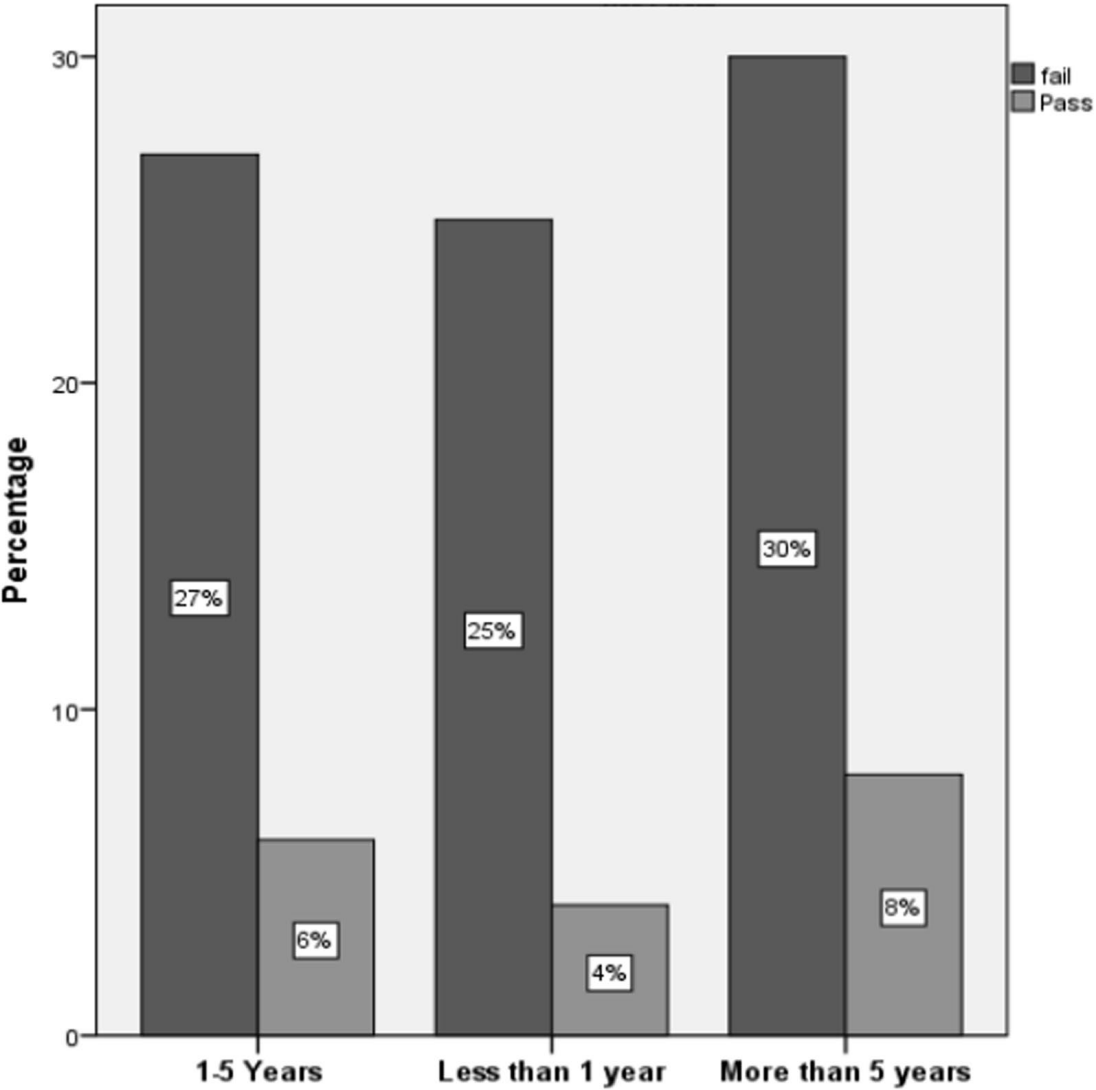
Providing appropriate counseling according to pharmacist working experience

The data presented in Fig. 3 clearly demonstrate that pharmacists who possess substantial work experience (exceeding 5 years) exhibited markedly higher rates of correct responses pertaining to patient counseling matters when compared to their counterparts with limited experience (less than 1 year), yielding correct answer percentages of 8% and 4%, respectively. This distinction in performance was found to be statistically significant (*P* < 0.05). In contrast, there were no statistically significant disparities between pharmacists with moderate experience (1–5 years) and those with minimal work experience (less than 1 year), as they both recorded correct answer rates of 6% and 4%, respectively. Comparable research focusing on pharmacist evaluations has consistently indicated that job experience plays a substantial role in enhancing pharmacists' knowledge [44, 45].

The results showed that there was variation among Palestinian cities. For example, pharmacists residing in the Ramallah district scored better than those residing in other cities. However, the difference was not statistically significant (*P* > 0.05); detailed results are presented in Table 4. The results demonstrate the lack of knowledge is almost equally distributed among the studied cities, which represent the northern and middle parts of the Palestinian territories. The result of this study is similar to the finding of Hussain et al., done in different cities of Pakistan, showing a lack of principal dispensing knowledge among Pakistani pharmacists in different cities [46].

**Table 4:** Pharmacist scores according to districts

District	Fail (%)	Pass (%)
Jenin	85	15
Nablus	95	5
Ramallah	60	40
Salfeet	95	5
Tulkarm	75	25

The overall findings highlight the critical need for more training in drug-food counseling. Radwan et al. found a lack of knowledge about food-drug interactions among local pharmacists in their study [47]. Many other studies demonstrate the need and desire of pharmacists for training in proper and correct patient counseling [48–50]. The responsibility lies with the pharmacy association as well as the ministry of health to make pharmacy education and pharmacist updating of knowledge compulsory for the renewal of licenses of practicing community and hospital pharmacists, as this is done in other neighboring and international countries.

### Study limitation

Increasing the sample size would enhance the study's generalizability, as it did not encompass interviews with hospital pharmacists or pharmacists employed in ministry of health clinical centers. Furthermore, the study did not address rural areas, primarily due to the limited presence of community pharmacies in these regions. Nevertheless, it is advisable for future research to incorporate this category into their investigations.

## Conclusion

In this research project, we conducted an assessment of pharmacists' competencies related to patient counseling, specifically focusing on advising patients regarding the timing of medication intake and imparting medication-related instructions. The findings revealed a concerning trend as the majority of the surveyed pharmacists provided incorrect responses to the evaluation questionnaire. This outcome sheds light on their limited understanding of drug interactions with food and suggests that they may be operating with outdated counseling knowledge. In light of these findings, we strongly advocate for the establishment of a continuous education system tailored to Palestinian pharmacists, especially those engaged in community pharmacy practice. Such a program would serve as a valuable resource to refresh and update their knowledge on the proper dispensation of medications, including pertinent details about drug interactions and counseling techniques. Furthermore, we propose that the Ministry of Health (MOH), in collaboration with pharmacy associations, assume responsibility for immediate and essential corrective measures. It is imperative to raise awareness among pharmacists about the critical role they play in providing accurate and thorough counseling to patients before dispensing medications. By doing so, we can ensure that patients receive safe and effective treatment while fostering a culture of continuous.

### Supplementary Information

**Additional file 1: **Assessment questionnaire for interviewed pharmacist.

## Data Availability

All data generated or analyzed during this study are included in this published article [and its Additional file 1.
