# Supplementary material for: Pharmacists' knowledge of drug food administration and their appropriate patient counseling a cross-sectional study from Palestine
Source: J Health Popul Nutr. 2023 Sep 14;42:99. doi: 10.1186/s41043-023-00444-9 (PMC10500721; doi:10.1186/s41043-023-00444-9)
Supplement: Supplementary file 1 — Additional file 1: Assessment questionnaire for interviewed pharmacist. [file 41043_2023_444_MOESM1_ESM.docx]

**Supplementary Material**

**Pharmacists' knowledge of drug food administration**

**and their appropriate patient counseling a cross sectional study from Palestine**

*Murad Abualhasan ^*1^, Shahd Tahan ^1^, Roa’a Nassar ^1^, Maysoon Damere ^1^, Hadeel Salameh ^1^ and Hiba Zyoud ^1^*

^1^ Department of Pharmacy, Faculty of Medicine and Health Sciences, An-Najah National University, P.O. Box 7, Nablus, State of Palestine

***Correspondence:** m_abualhasan@najah.edu

**(Questionnaire)**

Dear Pharmacist,

The main objective of this study is to stress on the importance and the rule of the community pharmacist instructions to their patient regarding administering of certain drugs .

| Quest Serial No: |
| --- |
| Interviewer ID: |
| District: |
| City/ village: |

| Age: |
| --- |
| Gender: |
| Years of experience: |
| Year of establishing Community pharmacy shop: |
| Degree: |
| Year of graduation: |
| Number of pharmacist in the shop: |
| Pharmacy manager/assistant pharmacist: |

**Questions:**

**1* Metformin**

a) Before meal

b) After meal

c)Can be taken both after or before meal

*Any necessary instruction:_______________________________________________________________

**2* Glibenclamide (Glucocare®)**

a) Before meal

b) After meal

c)Can be taken both after or before meal

*Any necessary instruction:_______________________________________________________________

**3* Acarbose (Prandase®)**

a) Before meal

b) After meal

c)Can be taken both after or before meal

*Any necessary instruction:_______________________________________________________________

**4* Cefuroxime(Zinnat)**

a) Before meal

b) After meal

c)Can be taken both after or before meal

*Any necessary instruction:_______________________________________________________________

**5* Azithromycin**

a) Before meal

b) After meal

c)Can be taken both after or before meal

*Any necessary instruction:_______________________________________________________________

**6* Ciprofloxacin (Ciprocare®)**

a) Before meal

b) After meal

c)Can be taken both after or before meal

*Any necessary instruction:_______________________________________________________________

**7* Fosfomycin Tromethamin (Monurol®)**

a) Before meal

b) After meal

c)Can be taken both after or before meal

*Any necessary instruction:___________________________________________________________

**8* Naproxen sodium**

A) Before meal

b) After meal

c)Can be taken both after or before meal

*Any necessary instruction:___________________________________________________________

**9* Diclofenac potassium (Cataflam®)**

a) Before meal

b) After meal

c)Can be taken both after or before meal

*Any necessary instruction:___________________________________________________________

**10* Celecoxib (Coxib®)**

a) Before meal

b) After meal

c)Can be taken both after or before meal

*Any necessary instruction:___________________________________________________________

**11* Sulindac (mobicol®)**

a) Before meal

b) After meal

c)Can be taken both after or before meal

*Any necessary instruction:___________________________________________________________

**12* Phenazopyridine(Utised®)**

a) Before meal

b) After meal

c)Can be taken both after or before meal

*Any necessary instruction:___________________________________________________________

**13* Clindamycin HCL (tab)**

a) Before meal

b) After meal

c)Can be taken both after or before meal

*Any necessary instruction:___________________________________________________________

**14* ulipristal acetate (ella® )**

a) Before meal

b) After meal

c)Can be taken both after or before meal

*Any necessary instruction:___________________________________________________________

**15* Isotretinoin(Curatane ®)**

a) Before meal

b) After meal

c)Can be taken both after or before meal

*Any necessary instruction:___________________________________________________________

**16* Spironolactone**

a) Before meal

b) After meal

c)Can be taken both after or before meal

*Any necessary instruction:___________________________________________________________

**17* Alendronate (Osteotab ®)**

a) Before meal

b) After meal

c)Can be taken both after or before meal

*Any necessary instruction:___________________________________________________________

**18* Levothyroxine (Euthyrox®)**

a) Before meal

b)After meal

c)Can be taken both after or before meal

*Any necessary instruction__________________________________________________

**19*Fluconazole (Dican®) (capsule)**

a) Before meal

b) After meal

c) Can be taken both after or before meal

*Any necessary instruction:____________________________________________________

**20* fluoxetine (Fluoxicare®)**

a) Before meal

b)After meal

c)Can be taken both after or before meal

*Any necessary instruction:___________________________________________________
